# From standard to stratified: Modeling NTCP and EAR to personalize daily MV‐CBCT in radiotherapy

**DOI:** 10.1002/acm2.70414

**Published:** 2025-12-10

**Authors:** Duong Thanh Tai, Luong Tien Phat, Tran Trung Kien, Duong Tuan Linh, Nguyen Ngoc Anh, Nguyen Quang Hung, Peter Sandwall, Parham Alaei, David Bradley, James C. L. Chow

**Affiliations:** ^1^ Department of Medical Physics Faculty of Medicine Nguyen Tat Thanh University Ho Chi Minh City Vietnam; ^2^ Department of Radiation Oncology University Medical Shing Mark Hospital Dong Nai Vietnam; ^3^ The Laboratory Center of Hanoi University of Public Health Hanoi University of Public Health Hanoi Vietnam; ^4^ Department of Applied Physics School of Engineering Sciences KTH Royal Institute of Technology Stockholm Sweden; ^5^ Phenikaa Institute for Advanced Study (PIAS) Phenikaa University Hanoi Vietnam; ^6^ Institute of Fundamental and Applied Sciences Duy Tan University Ho Chi Minh City Viet Nam; ^7^ Faculty of Natural Sciences Duy Tan University Da Nang Viet Nam; ^8^ Department of Radiation Oncology OhioHealth Mansfield Mansfield Ohio USA; ^9^ Department of Radiation Oncology University of Minnesota Minneapolis Minnesota USA; ^10^ Applied Radiation Physics and Technologies Group Department of Engineering Sunway University Petaling Jaya Selangor Malaysia; ^11^ School of Mathematics and Physics University of Surrey Guildford Surrey UK; ^12^ Department of Radiation Oncology University of Toronto Toronto ON Canada; ^13^ Radiation Medicine Program, Princess Margaret Cancer Centre University Health Network Toronto ON Canada

**Keywords:** EAR, Halcyon platform, imaging dose, imaging protocol, MV‐CBCT, NTCP, radiobiological modeling, secondary cancer risk

## Abstract

**Purpose:**

To evaluate the cumulative radiobiological impact of daily megavoltage cone‐beam computed tomography (MV‐CBCT) imaging dose based on normal tissue complication probability (NTCP) and excess absolute risk (EAR) of secondary malignancies among radiotherapy patients treated for breast, pelvic, and head & neck cancers. This study investigated whether MV‐CBCT imaging dose warrants protocol personalization according to patient age, anatomical treatment site, and organ‐specific radiosensitivity.

**Methods:**

This retrospective study included cohorts of breast (*n* = 30), pelvic (*n* = 17), and head & neck (*n* = 20) cancer patients undergoing radiotherapy with daily MV‐CBCT. Imaging dose distributions employing two common MV‐CBCT protocols (5 and 10 MU per fraction) were analyzed. NTCP values were estimated using logistic models, while EAR were calculated using Schneider's organ equivalent dose (OED)‐based model, integrating organ‐specific dose, patient age, and established tissue‐specific risk coefficients. Comparative statistical analyses were conducted using paired *t*‐tests, and results were further stratified by patient age (< 40, 40–60, > 60 years).

**Results:**

In breast cancer patients, NTCP values increased significantly for lung tissue when comparing the 10 MU protocol to the 5 MU one (*p* < 0.001), while those for heart and breast tissues showed minimal and insignificant differences. EAR estimations revealed substantial risk increases among younger breast cancer patients (< 40 years), with some exceeding 15 cases per 10 000 person‐years under the 10 MU protocol. Conversely, pelvic and head & neck cohorts demonstrated consistently low NTCP and EAR values (< 1%), with no meaningful differences observed between the two imaging protocols. Across all cancer sites, younger age consistently correlated with higher secondary cancer risks.

**Conclusion:**

Routine daily MV‐CBCT imaging at the 10 MU protocol possesses minimal additional risk in pelvic and head & neck radiotherapy. However, among breast cancer patients, particularly those under 40 years, the 10 MU protocol significantly elevates the theoretical secondary cancer risk estimates and lung NTCP. These findings support transitioning from conventional uniform imaging approach toward personalized MV‐CBCT protocols, tailored according to patient age, anatomical site, and organ radiosensitivity. A stratified imaging framework is proposed to optimize clinical outcomes, balancing treatment accuracy, and long‐term patient safety.

## 1 INTRODUCTION

Image‐guided radiotherapy (IGRT) has revolutionized radiation oncology by leveraging medical imaging to enhance the precision of radiation delivery, ensuring optimal tumor targeting while sparing healthy tissues.^1^, ^2^, ^3^, ^4^, ^5^, ^6^ Megavoltage cone‐beam computed tomography (MV‐CBCT), a key IGRT modality, uses the treatment beam to generate three‐dimensional anatomical images, enabling daily verification of patient positioning.^7^, ^8^ Integrated into platforms like the Varian Halcyon (Varian Medical Systems, Palo Alto, CA, USA), MV‐CBCT offers two standard imaging protocols, “Low Dose” (5 monitor units [MU]) and “High Quality” (10 monitor units [MU]), balancing image quality with radiation exposure.^9^, ^10^ These protocols are critical for verifying accurate setups yet their routine use raises concerns about cumulative imaging doses, particularly in long‐term cancer survivors.^11^, ^12^ The eclipse treatment planning system (TPS, Varian Medical Systems, Palo Alto, CA, USA), is employed for treatment planning and dose calculations on the Halcyon platform, allowing for accurate computation of imaging dose contributions using algorithms identical to those utilized in therapeutic dose calculations. This capability facilitates the incorporation of MV‐CBCT dose into the total dose patient receives.

Numerous studies on the quantification of organ doses from MV‐CBCT have reported that while individual fractions deliver low doses, cumulative exposure over multiple fractions can be significant, especially for radiosensitive organs.^12^, ^13^ Efforts to mitigate such a cumulative exposure include optimizing CBCT protocols, adopting low‐dose modes, and following guidelines from AAPM Task Groups (TG‐75 and TG‐180) for imaging dose quantification and integration into clinical planning.^14^, ^15^, ^16^ Despite these advances, the cumulative biological impact of MV‐CBCT, including risks of late normal tissue complications and secondary malignancies, remain underexplored.^17^, ^18^, ^19^, ^20^ Recent modeling studies have suggested that secondary cancer risks depend on age, imaging frequency, and anatomical site.^21^, ^22^ However, a systematic application of radiobiological models, such as NTCP for toxicity and EAR for secondary cancer risk, is not available. Meanwhile, current MV‐CBCT protocols often adopt a uniform approach, disregarding patient‐specific factors like age or organ radiosensitivity. This “one‐size‐fits‐all” strategy may lead to unnecessary radiation exposure, particularly in younger patients, who face elevated lifetime risks due to higher cellular sensitivity and longer survival.^11^ In particular, while expert consensus recommends imaging dose calculations using planning CT datasets to mitigate CBCT image artifacts, tailored imaging protocols based on patient demographics and anatomical variations are rarely implemented.

This study addresses the aforementioned gaps by systematically evaluating the dosimetric and radiobiological effects of daily MV‐CBCT imaging in breast, pelvic, and head & neck radiotherapy. Using established models, including logistic and Lyman–Kutcher–Burman (LKB) NTCP models and Schneider's organ equivalent dose (OED)‐based EAR method, we quantify the theoretical estimated risks of toxicity and secondary cancers associated with standard MV‐CBCT protocols (5 and 10 MU). Our specific aims are: (1) to assess NTCP and EAR across three treatment sites; (2) to examine age‐dependent variations in secondary cancer risk; and (3) to propose evidence‐based, personalized MV‐CBCT imaging strategies that account for patient age, anatomical site, and organ‐specific radiosensitivity. By integrating rigorous dosimetry with advanced radiobiological modeling, this research seeks to inform clinicians, thus promoting individualized IGRT protocols that maintain treatment precision while minimizing long‐term risks.

## 2 MATERIALS AND METHODS

**FIGURE 1:**
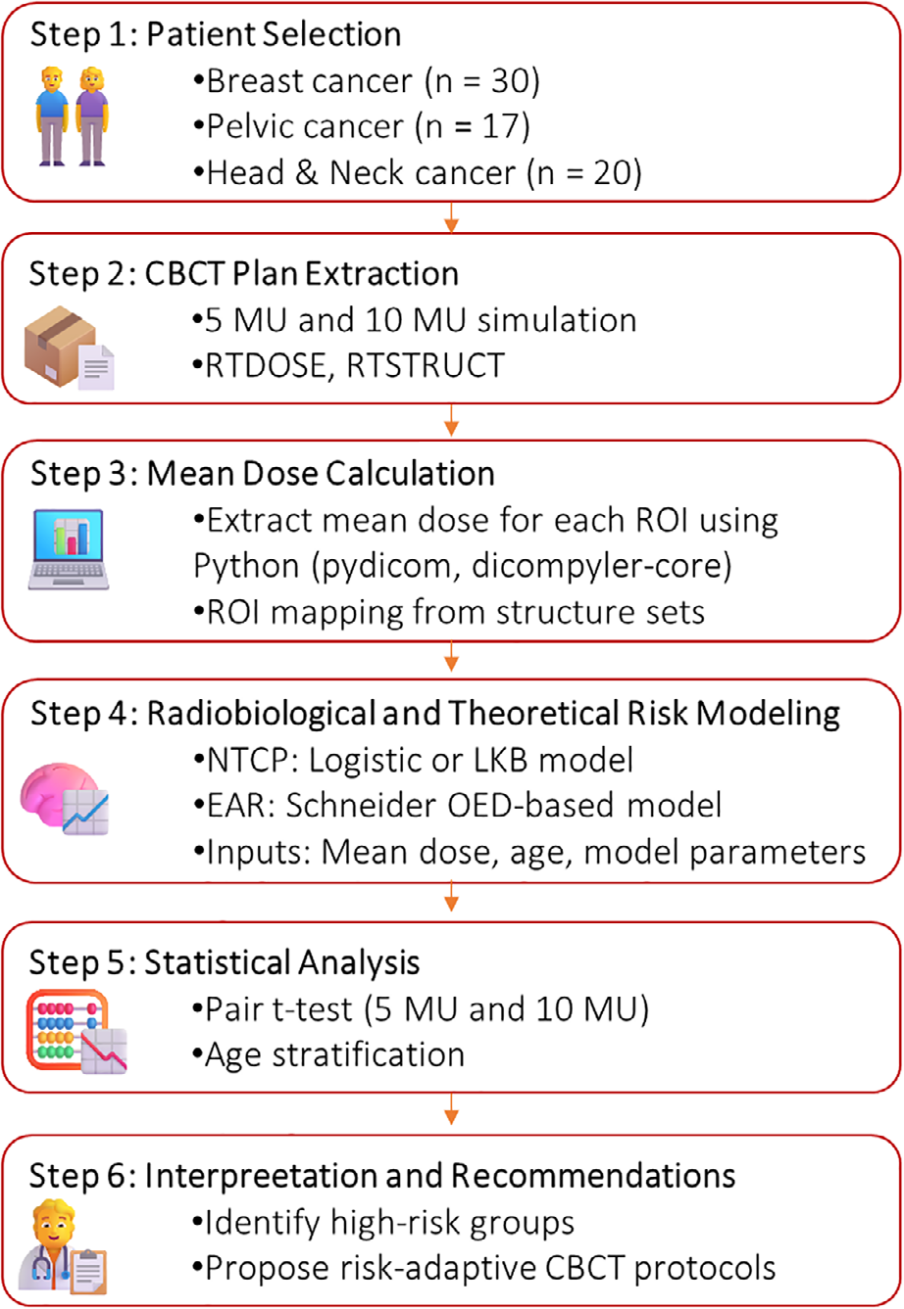
Workflow of CBCT dose analysis and NTCP & EAR modeling techniques.

This study evaluates the imaging dose associated with MV‐CBCT in radiotherapy for three cancer sites, following a structured workflow to assess its dosimetric and potential radiobiological impacts. The methodology is detailed in Figure 1, encompassing patient selection, data extraction, dose calculation, risk modeling, statistical analysis, and interpretation strategy.

### 2.1 Patient selection

Our retrospective study involved 67 patients who underwent definitive radiotherapy (*n* = 30), pelvic (*n* = 17), and head and neck (*n* = 20) cancers. Inclusion criteria required patients to be aged ≥18 years, who have complete DICOM datasets (planning CT, RTSTRUCT, RTPLAN, RTDOSE), and receive daily MV‐CBCT for setup verification using the Halcyon linear accelerator. Exclusion criteria included incomplete imaging records or prior radiotherapy to the same anatomical site. The study was approved by the hospital's ethics committee. Patient demographics, including age and sex, are summarized in Table S1.

Two MV‐CBCT imaging protocols were simulated for each patient, namely low‐dose (5 MU per fraction) and high‐dose (10 MU per fraction) protocols. These protocols were modeled over 25 fractions for breast, 28 fractions for pelvic, and 35 fractions for head and neck cancers, reflecting typical clinical schedules. MV‐CBCT acquisition involved a gantry rotation from 260° to 100°, consistent with Halcyon system specifications.

### 2.2 CBCT plan and extraction

MV‐CBCT imaging dose plans were generated in Eclipse (v15.5) using the analytic anisotropic algorithm (AAA), incorporating the Fourier transform dose calculation (FTDC) module for improved accuracy. To isolate CBCT doses, beam doses in the treatment plan were set to zero.^16^ Daily MV‐CBCT images were rigidly registered to the planning CT in Eclipse to map dose grids to organs at risk (OARs) (e.g., heart, lungs, rectum, parotid glands).

The AAA was selected because it is the clinically commissioned and vendor‐validated model for the Halcyon MV‐CBCT beam within Eclipse at our institution. The Acuros XB (AXB) algorithm, although more advanced for treatment planning, currently lacks a validated MV‐CBCT beam model on the Halcyon platform. Previous studies have confirmed that AAA provides comparable accuracy (within ± 2%) to AXB for dose calculations on CBCT datasets across different anatomical sites, including heterogeneous regions such as the lung and head and neck ^23^, ^24^, ^25^

DICOM data, including RTSTRUCT and RTDOSE files, were processed using a validated Python‐based pipeline developed with pydicom (v2.4.4) and dicompyler‐core (v0.7.1). Dose distributions for both 5 and 10 MU protocols from a single fraction were extracted, and accumulated over the full treatment course. Final dose matrices were cross‐validated against Eclipse exports to ensure data integrity.

### 2.3 Mean dose calculation

Mean doses to regions of interest (ROIs) were calculated using Python‐based tools, specifically pydicom for DICOM handling and dicompyler‐core (v0.7.1) for dose‐volume analysis. ROIs were defined based on clinical contouring guidelines for each cancer site, including OARs such as the heart, lungs, bladder, rectum, brainstem, optic nerves, and parotid glands, as specified in the RTSTRUCT files. ROI mapping was performed to standardize nomenclature across patients, ensuring consistency in dose reporting. The dose grids from RTDOSE files were overlaid onto the corresponding ROIs, and the mean dose per ROI was computed as the arithmetic average of dose values within the contoured volume. All calculations were performed in a Python environment (version 3.11) to ensure reproducibility.

### 2.4 Radiobiological modeling

Normal tissue complication probability (NTCP) was modeled using the logistic model.^26^

(1) $$NTCP=\frac{1}{1+e^{\gamma \left(D_{50}-D\right)}}\;$$

NTCP was calculated using mean dose and organ‐specific parameters (*D*_50_, *m*, *n*, or *γ*), as summarized in Table S2. These parameters were selected from peer‐reviewed literature with established clinical relevance. Model computations were implemented in Python using *numpy* and *scipy* libraries.

NTCP values supported comparison of subclinical complication risk between 5 and 10 MU CBCT protocols across cancer sites.

### 2.5 Secondary cancer risk estimation

The potential development of secondary cancers is an important concern in long‐term survivors undergoing CBCT‐guided radiotherapy. This study assessed the theoretical secondary cancer risk associated with MV‐CBCT imaging using Schneider's organ equivalent dose (OED)‐based model,^27^ which integrates dosimetric and radiobiological parameters. The methodology comprised two main steps: calculating the OED from organ‐specific differential dose‐volume histograms (dDVHs), and subsequently determining the EAR of secondary malignancy development.^28^, ^29^

#### 2.5.1 Organ equivalent dose (OED) calculation

The OED for each organ‐at‐risk (OAR) was calculated according to Schneider's formalism as follows:

(3) $$OED=\frac{1}{V}\;\sum_{i}D_{i}.e^{-\alpha^{\prime }D_{i}}$$

where $D_{i}\;$ is the dose to voxel *i*, *V* is the total organ volume, and *α*′ = 0.085 represents the cell sterilization parameter reflecting tissue radiosensitivity.

#### 2.5.2 Excess absolute risk (EAR) calculation

The EAR quantifies the additional cancer cases attributable to radiation exposure per 10 000 person‐years (PY). It was calculated using the following relationship:^30^

(4) $$EAR=EAR_{0}\;.OED.\mu \left(e,a\right)$$

where *EAR*_0_ is the baseline risk per Sv (organ‐specific, from BEIR VII),^31^, ^32^ and μ(e,a) where *e* is age at exposure and *a* is attained age (fixed at 70). Modeling parameters are listed in Table S3. All calculations were performed in Python using numpy (v1.26.4) and scipy (v1.13.1).

**Stratification by age**: Patients were grouped into three age categories (< 40, 40–60, > 60 years old) to examine age‐related differences in secondary cancer risk. This stratification was based on radiobiological evidence showing that the EAR for radiation‐induced secondary cancers typically decreases with increasing age at exposure.^31^ The < 40 group represents younger patients with longer life expectancy and potentially higher radiosensitivity, while the > 60 group typically faces lower lifetime cancer risk from radiation exposure.

Model parameters for each OAR are listed in Table S3. EAR values were reported in units of cases per 10 000 person‐years. All computations were performed using Python (numpy v1.26.4, scipy v1.13.1).

### 2.6 Statistical analysis

Dose metrics (mean dose), NTCP, and EAR were compared between 5 and 10 MU protocols using two‐tailed paired *t*‐tests. Age‐stratified analysis was conducted across three groups: < 40 years, 40–60 years, and > 60 years. Statistical significance was set at *p* < 0.05.

Data processing and statistical computations were conducted in Python (v3.9), with visualizations generated using matplotlib‐based boxplots. Results are reported as mean  ±  standard deviation, and references to Tables S4–S7 are provided where applicable.

### 2.7 Interpretation strategy

Results from the NTCP and EAR analyses were used to identify theoretical high‐risk patient subgroups, particularly focusing on age‐dependent sensitivity. These findings guided the development of personalized CBCT protocol recommendations, with the aim of reducing unnecessary imaging dose while preserving setup accuracy. All NTCP and EAR calculations in this study represent estimated theoretical risks derived from established radiobiological models, rather than clinical observations.

## 3 RESULTS

This section evaluates the impact of daily MV‐CBCT imaging on NTCP and EAR across three cancer sites: breast, pelvic, and head & neck. The analysis compares two imaging protocols: 5 and 10 MU. All reported values represent the total accumulated dose, including both the treatment beam and daily MV‐CBCT imaging contributions.

### 3.1 Breast cancer (*n* = 30)

#### 3.1.1 Imaging dose to OARs

A consistent and statistically significant dose escalation was observed when comparing 5 to 10 MU protocols over 25 fractions. For the contralateral breast region (Breast_CNTR), the mean dose increased from 3.93  ±  0.46 to 4.65  ±  0.46 Gy. Similarly, for the contralateral lung (Lung_CNTR), the corresponding dose increased from 3.91  ±  0.44 Gy (5 MU) to 4.66  ±  0.50 Gy (*p* < 0.001) (10 MU), while the heart dose rose from 6.89  ±  2.01 Gy (5 MU) to 7.90  ±  2.05 Gy (*p* < 0.001) (10 MU). The ipsilateral lung (Lung_IPSI) received the highest mean dose, increasing from 18.77  ±  0.78 Gy (5 MU) to 19.86  ±  0.78 Gy (*p* < 0.001) (10 MU).

**FIGURE 2:**
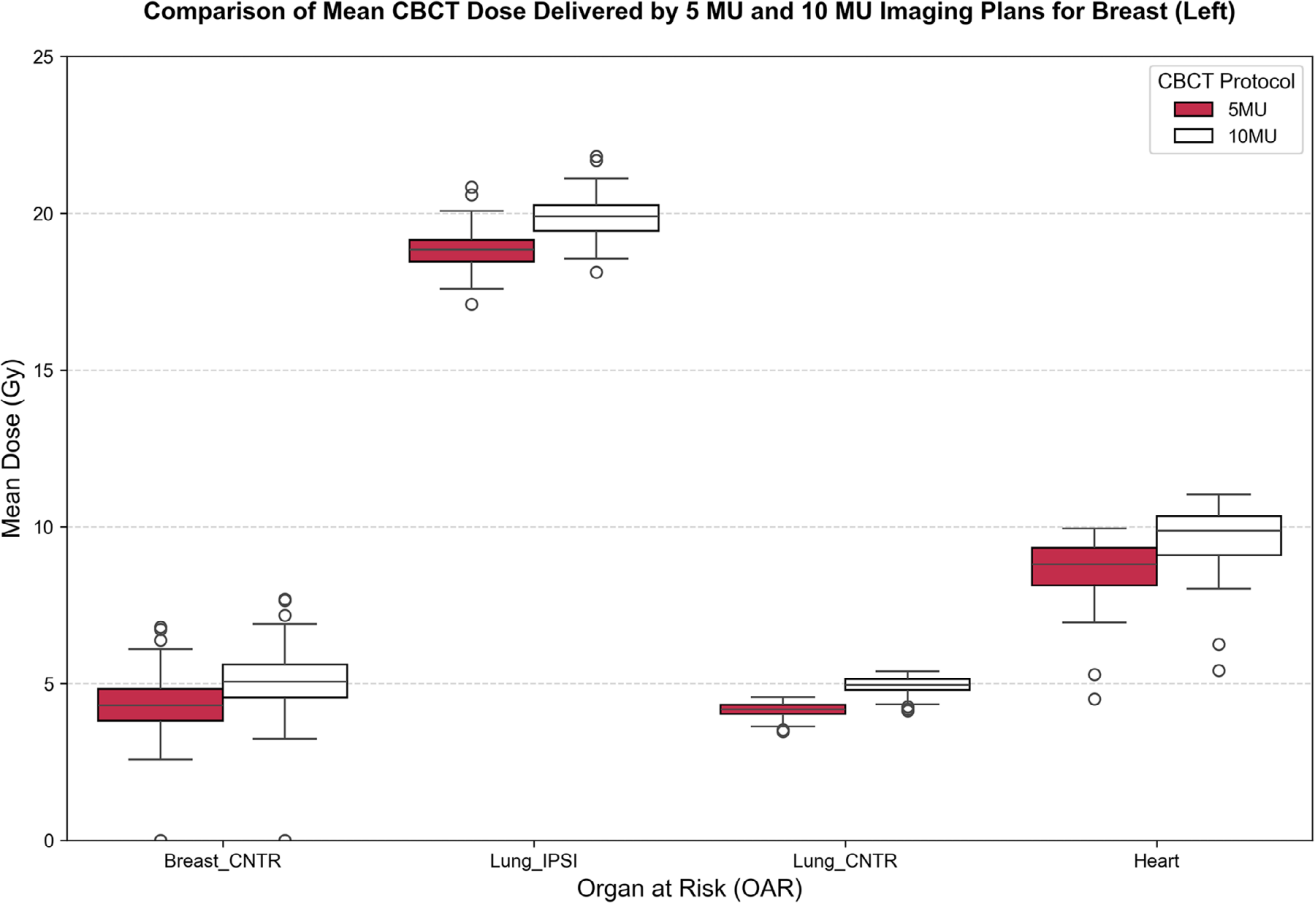
Boxplot of mean‐dose comparison between 5 and 10 MU CBCT protocols over 25 fractions for right‐sided breast cancer patients.

**FIGURE 3:**
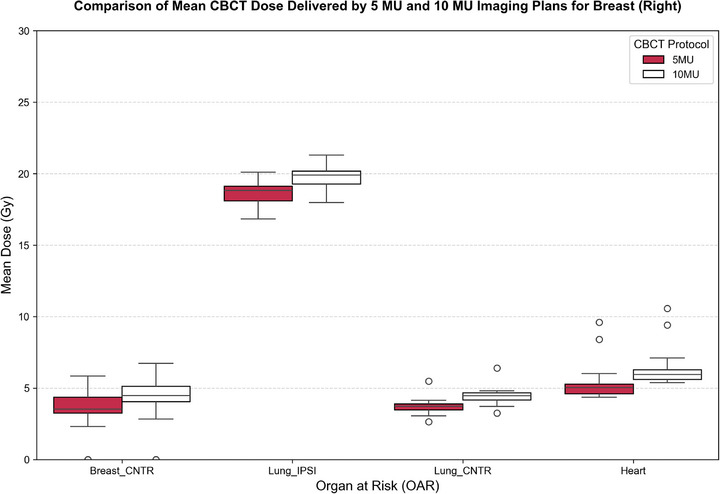
Boxplot of mean dose comparison between 5 and 10 MU CBCT protocols over 25 fractions for left‐sided breast cancer patients.

These findings are visualized in Figures 2 and 3, which present boxplots for right‐ and left‐sided breast cancer patients, respectively. Table 1 provides detailed dosimetric values and statistical comparisons. On average, dose increments ranged between 25% and 30% across all structures.

**TABLE 1:** Mean dose for breast cancer patients under 5 and 10 MU CBCT protocols over 25 fractions.

ROI	Dose (5 MU) Mean ± SD (Gy)	Dose (10 MU) Mean ± SD (Gy)	*p*‐value
Breast_CNTR	3.93 ± 1.46	4.65 ± 1.63	<0.001
Heart	6.89 ± 2.01	7.90 ± 2.05	<0.001
Lung_CNTR	3.91 ± 0.44	4.66 ± 0.50	<0.001
Lung_IPSI	18.77 ± 0.78	19.86 ± 0.78	<0.001

#### 3.1.2 NTCP analysis

As summarized in Table 2, NTCP values for all evaluated OARs remained well below 1%, indicating minimal clinical risk. However, statistically significant increases were observed for both lungs under the 10 MU protocol. Specifically, NTCP for Lung_IPSI increased from 0.00261% to 0.00317% (*p* < 0.001), and for Lung_CNTR from 0.00246% to 0.00283% (*p* < 0.001). No significant changes were noted for the heart or Breast_CNTR.

**TABLE 2:** NTCP for breast cancer patients under 5 and 10 MU CBCT protocols.

ROI	NTCP (5 MU) [%]	NTCP (10 MU) [%]	*p*‐value
Breast_CNTR	# ± #	# ± #	NS
Heart	# ± #	# ± #	NS
Lung_CNTR	0.00246 ± 0.00005	0.00283 ± 0.00010	<0.001
Lung_IPSI	0.00261 ± 0.00004	0.00317 ± 0.00009	<0.001

*Note*: The # sign defines that the value is close to zero.

Abbreviation: NS, not significant.

These results suggest that although absolute NTCP values are low, imaging dose escalation may incrementally increase pulmonary complication probability.

#### 3.1.3 Estimated theoretical excess absolute risk (EAR)

**FIGURE 4:**
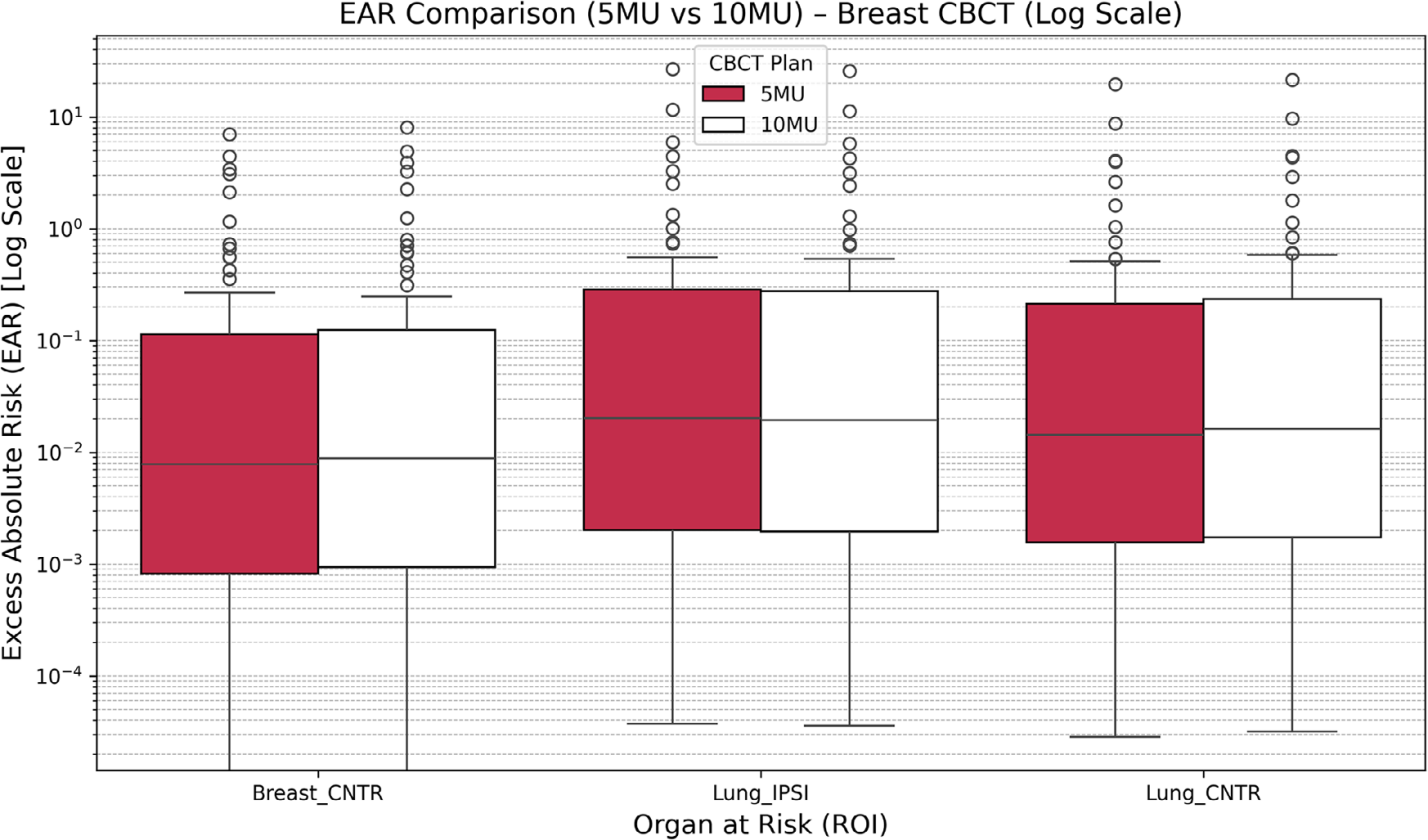
EAR distribution (log scale) for breast cancer patients comparing 5 vs. 10 MU CBCT plans. Boxplots illustrate the EAR for the Breast_CNTR, Lung_IPSI, Lung_CNTR, and Heart. The EAR values are presented in logarithmic scale to highlight interquartile variability and potential outliers.

Figure 4 illustrates EAR distributions for Breast_CNTR, Lung_IPSI, Lung_CNTR, and Heart across both protocols on a logarithmic scale. The 10 MU protocol resulted in significant EAR increases across all OARs (*p* < 0.001). For instance, EAR for Lung_IPSI doubled from 6.4  ±  3.1 to 12.8  ±  5.9 cases/10 000 person‐years (PY), while Breast_CNTR rose from 5.9  ±  2.7 to 11.2  ±  4.8 cases/10 000 PY.

Age‐stratified analysis (Table S4) revealed that patients under 40 exhibited the highest susceptibility, with EAR exceeding 15 cases/10 000 PY in both Breast_CNTR and Lung_IPSI under the 10 MU protocol. The maximum recorded EAR reached 672.9 × 10^−^⁴ PY (6.7 cases/1,000 PY) in a 30‐year‐old patient. Patients aged 40–60 showed moderate risks (∼7.1–18.0 × 10^−^⁴ PY), while those over 60 exhibited negligible risk (EAR < 0.05 × 10^−^⁴ PY).

These findings highlight a strong inverse correlation between age and secondary cancer risk, reinforcing the importance of tailoring imaging dose protocols based on patient age.

### 3.2 Pelvic cancer (*n* = 22)

#### 3.2.1 Imaging dose to OARs

Dosimetric analysis demonstrated a consistent and statistically significant increase in mean dose to pelvic OARs when shifting from the 5 to the 10 MU CBCT protocol. The rectum received the highest exposure, with mean dose rising from 48.71 ± 10.15 to 49.62 ± 10.32 Gy (*p* < 0.001). The bladder dose increased from 36.22 ± 4.12 to 37.41 ± 4.21 Gy (*p* < 0.001), and the bowel bag followed a similar pattern, from 25.47 ± 3.78 to 26.53 ± 3.89 Gy (*p* < 0.001).

**FIGURE 5:**
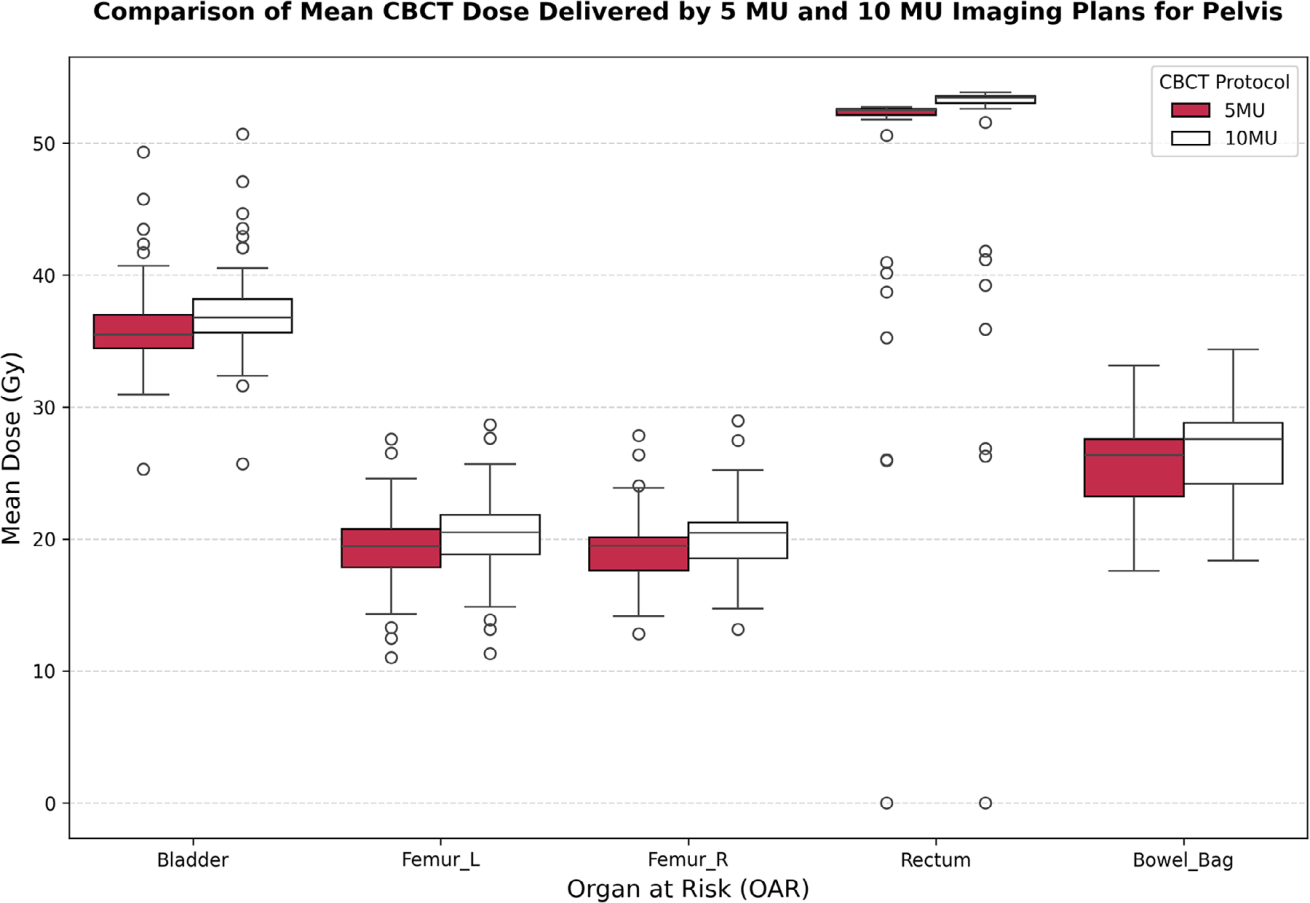
Boxplot of mean dose comparison between 5 and 10 MU CBCT protocols over 28 fractions for pelvic cancer patients.

These differences are visualized in Figure 5, which shows the distribution of mean dose across OARs under both CBCT protocols. The upward shift in dose levels under 10 MU is consistent across all evaluated pelvic structures.

#### 3.2.2 NTCP analysis

Although NTCP values remained below 0.5% across all pelvic OARs, the 10 MU protocol yielded statistically significant increases. The rectum exhibited the greatest NTCP rise (0.0018% to 0.0042%, *p* = 0), with similarly small yet significant increases observed for the bladder and bowel bag. While these changes may not reach clinical thresholds, they suggest cumulative dose effects from daily imaging.

#### 3.2.3 Estimated theoretical excess absolute risk (EAR)

EAR values approximately doubled under the 10 MU protocol. EAR increased from 1.42  ±  0.59 to 2.88  ±  1.11 cases/10 000 PY for the rectum; from 1.26  ±  0.51 to 2.63  ±  1.03 cases/10 000 PY for the bladder; and from 1.13  ±  0.47 to 2.29  ±  0.92 cases/10 000 PY for the bowel.

Patients younger than 40 exhibited the highest EAR values, with rectum and bladder EARs exceeding 3 cases/10 000 PY. In contrast, patients over 60 showed negligible risk increases. Table A6 presents detailed ROI‐based EAR metrics.

**FIGURE 6:**
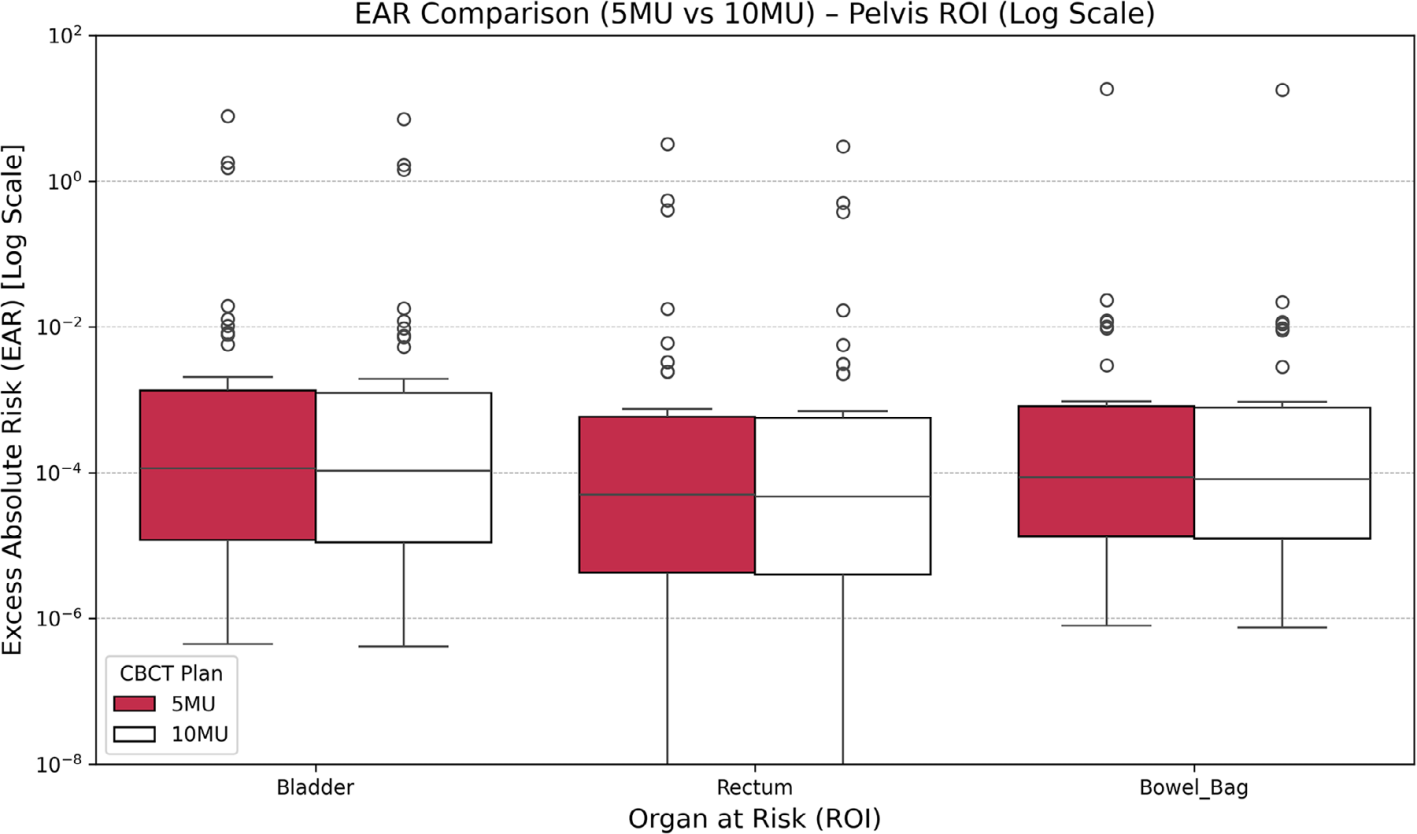
EAR distribution (log scale) for pelvic cancer patients comparing 5 vs. 10 MU CBCT plans. Boxplots display the EAR for the Bladder, Bowel_Bag, and Rectum. Results are shown on a logarithmic scale to emphasize the spread of EAR values across CBCT doses. The 10 MU group tends to have slightly higher EAR, though absolute values remain low.

Figure 6 visualizes EAR distributions (log scale), demonstrating a consistent rightward shift under the 10 MU protocol.

### 3.3 Head & neck cancer (*n* = 20)

#### 3.3.1 Imaging dose to OARs

**FIGURE 7:**
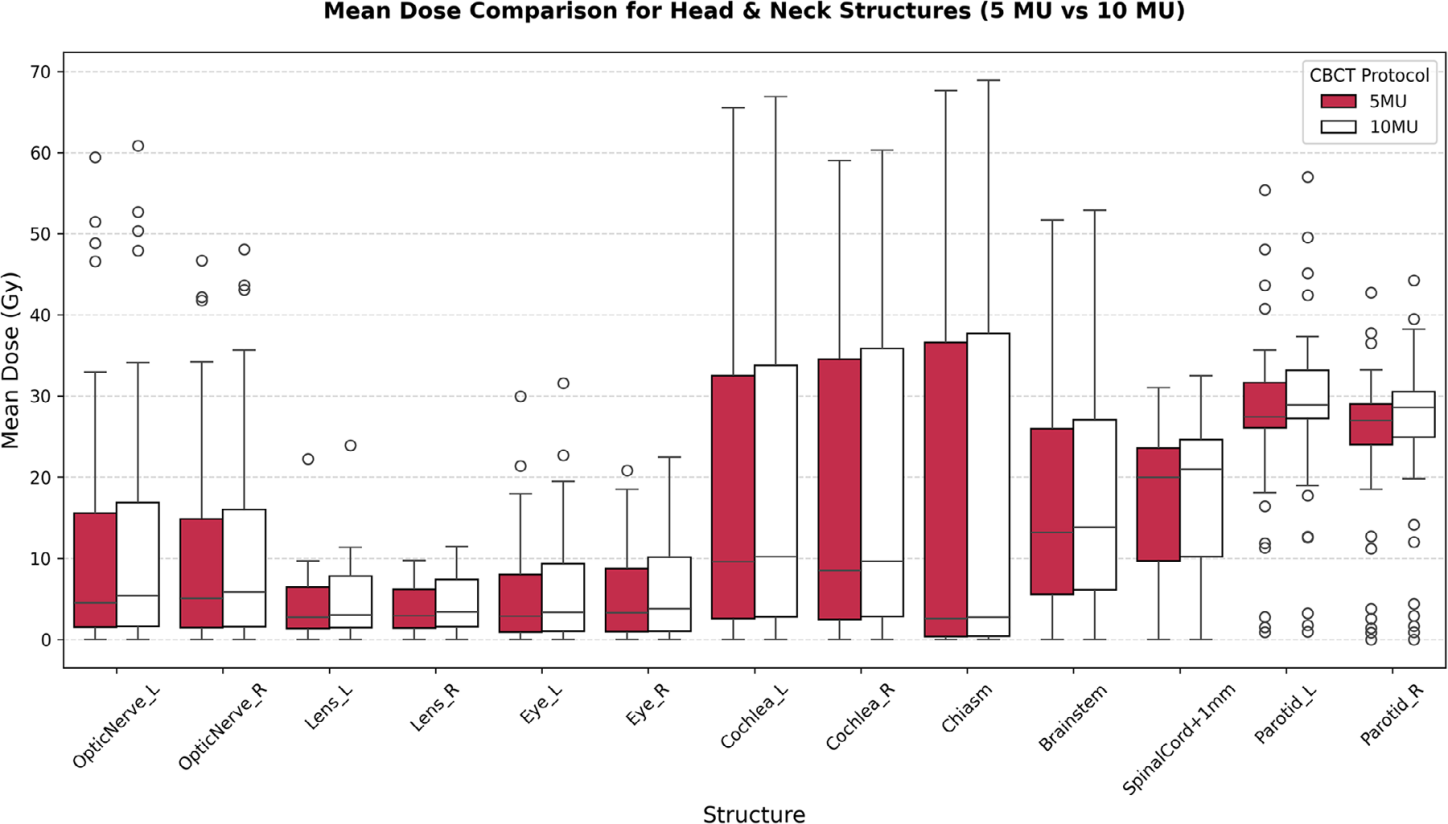
Boxplot of mean dose comparison between 5 and 10 MU CBCT protocols over 35 fractions for head & neck cancer patients.

CBCT protocols using 10 MU resulted in significantly higher mean doses across all examined OARs in head and neck cancer patients as compared to those using 5 MU (Figure 7). The most pronounced increases were observed in the parotid glands, spinal cord, brainstem, and optic nerves. For example, the spinal cord received a mean dose of 16.13  ±  9.99 Gy under 5 MU, increasing to 16.89  ±  10.42 Gy at 10 MU (*p*  <  0.001). The brainstem showed a similar trend, increasing from 16.00  ±  12.86 to 16.73  ±  13.21 Gy. Parotid doses increased from 26.98  ±  11.06 to 28.33  ±  11.38 Gy (left) and 24.19  ±  10.38 to 25.49  ±  10.80 Gy (right). All dose increases across ROIs were statistically significant (*p*  <  0.001). Figure 7 illustrates the dose escalation across major OARs between 5 and 10 MU protocols, confirming consistent dose elevation across all structures.

#### 3.3.2 NTCP analysis

Despite absolute NTCP values remaining < 1% across all head and neck OARs, a significant relative increase was observed with the 10 MU protocol. The spinal cord NTCP increased from 0.0008% to 0.0023%, while parotid glands showed the highest increase: from 0.0024% to 0.0067% (left) and from 0.0021% to 0.0062% (right), both *p*  <  0.001. These results, though small in magnitude, indicate heightened subclinical complication risks with increased imaging dose. NTCP results are further detailed in Table S2, which summarizes model parameters used for all OARs.

#### 3.3.3 Estimated theoretical excess absolute risk (EAR)

**FIGURE 8:**
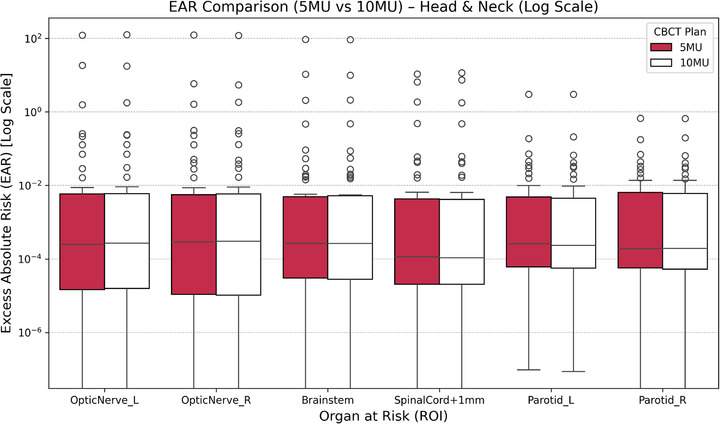
Boxplots illustrating the EAR for six OARs in head and neck cancer patients imaged using 5 and 10 MU CBCT protocols. EAR values are shown on a logarithmic scale for optic nerves (L/R), brainstem, spinal cord +1 mm, and parotid glands (L/R). The 10 MU protocol consistently resulted in higher EAR across all ROIs, with the largest differences observed in spinal cord and parotid glands. Overall, EAR values remained below 10^−^^2^ cases per 10 000 person‐years.

As shown in Figure 8, EAR values increased substantially under 10 MU, particularly in parotid glands and spinal cord. The highest EAR was observed in parotid glands, increasing from 1.33  ±  0.52 to 2.85  ±  1.08 (left) and from 1.27  ±  0.49 to 2.69  ±  1.02 (right). Brainstem and spinal cord EARs were lower (<1.2), but still approximately doubled under 10 MU. Age‐stratified analysis revealed that patients under 40 consistently exhibited higher EAR values across all structures, underscoring the greater susceptibility of younger individuals to second malignancies induced by CBCT exposure. A detailed breakdown of EAR for each ROI is presented in Table A7.

Figure 8 presents the EAR distribution on a logarithmic scale, clearly depicting the interquartile spread and the marked increase in EAR under the 10 MU protocol, especially in the parotid glands and spinal cord.

## 4 DISCUSSION

This study revealed that daily MV‐CBCT imaging significantly increases cumulative organ doses, with the 10 MU protocol yielding higher NTCP and EAR compared to the 5 MU one across breast, pelvic, and head & neck radiotherapy cohorts. These findings underscore the importance of optimizing imaging protocols to minimize long‐term radiobiological risks while maintaining treatment accuracy.

Our dosimetric results align with prior studies reporting that individual MV‐CBCT imaging deliver doses of several centigray (cGy) per fraction, with cumulative effects becoming notable over multiple fractions.^7^, ^8^, ^12^, ^13^, ^33^, ^34^ For instance, our mean doses for breast cancer OARs (e.g., 0.76–1.02 Gy for 5 MU; 1.21–1.98 Gy for 10 MU) are consistent with Malajovich et al. (2019), who reported similar organ doses for the Halcyon platform.^12^ However, those studies focused on physical dosimetry without evaluating clinical outcomes such as NTCP or secondary cancer risk. In contrast, our work integrates dosimetric data with NTCP and EAR models, providing a deeper understanding of the biological implications. Yuasa et al. ^21^ also modeled EAR in the context of 4D CBCT but their work was limited to thoracic imaging. Our study expands the scope by comparing three anatomical sites with age‐stratified risk, thereby offering a broader framework for risk‐informed protocol optimization.

The clinical implications of our findings are noteworthy. Although NTCP values remained low across all organs (typically <1%), significant relative increases were observed with the 10 MU protocol, particularly in lung and parotid tissues. This may translate to higher subclinical toxicity over time, especially in younger patients with long survival expectations. EAR values also increased substantially under the 10 MU protocol, with the most prominent changes seen in breast and head & neck cohorts. Our age‐stratified EAR analysis revealed up to a 30‐fold increase in theoretical secondary cancer risk among patients <40 years old versus those >60, supporting the need for age‐based imaging customization. These findings align with concerns raised by Hall and Wuu ^17^ and Kim et al.^18^ regarding imaging‐induced carcinogenesis.

Our findings also support current AAPM guidelines (TG‐75 and TG‐180) advocating dose reduction where possible,^14^, ^16^ and further underscore the utility of incorporating MV‐CBCT dose into total treatment planning. Eclipse's built‐in imaging dose calculation module allows for this integration, enabling biologically informed treatment reviews, as previously suggested by Murphy et al.^14^ Nevertheless, our findings suggest that the current “one‐size‐fits‐all” approach to CBCT imaging may lead to unnecessary long‐term radiation‐induced cancer risks, especially in younger patients. Given the sharply elevated EAR observed in patients under 40, a risk‐adaptive CBCT approach may be appropriate for this group. Specifically, lower‐dose imaging protocols (e.g., 5 MU) should be considered when image quality is clinically sufficient to ensure accurate setup. For patients aged 40–60, 5 MU protocols may still be suitable, provided that adequate image guidance is maintained for the anatomical site. In contrast, for patients over 60, current daily 10 MU protocols appear justifiable, as the long‐term risk of secondary cancer is minimal. These findings support the implementation of age‐informed CBCT strategies that balance image quality and patient‐specific radiobiological risk in routine clinical radiotherapy.

Despite its contributions, this study has limitations. The retrospective design and moderate sample size (*n* = 67) may limit generalizability, particularly for pelvic cancers (*n* = 17). The reliance on literature‐derived NTCP and EAR parameters introduces potential uncertainties, as inter‐patient radiosensitivity varies. According to the UNSCEAR 2012 Report (Annex B),^35^ uncertainties in radiation‐induced cancer risk modeling may vary by at least a factor of three, and in some organs by more than an order of magnitude. In addition, variations in the choice of radiobiological parameters (e.g., *α*/*β* ratios, *D*_50_, and risk coefficients) and model selection have been shown to significantly influence NTCP and secondary cancer risk predictions, with both aleatoric and epistemic uncertainties remaining intrinsic to such modeling and requiring explicit acknowledgment when interpreting theoretical estimates.^36^, ^37^, ^38^ This reflects the limited precision in current risk coefficients and dose–response models. Additionally, dose calculations based on planning CT, while recommended to mitigate CBCT artifacts, may introduce minor registration errors.^13^ The assumption of uniform imaging frequency across fractions may not reflect real‐world variations in clinical practice.

Future research should address these limitations through prospective studies with larger, more diverse cohorts to validate our findings. Developing adaptive CBCT protocols that adjust imaging dose and frequency based on patient age, tumor site, and organ radiosensitivity could further optimize the risk–benefit balance. Additionally, incorporating genomic data to refine radiobiological models could enhance the precision of NTCP and EAR predictions.

In summary, our findings advocate for the personalization of CBCT imaging strategies in radiotherapy. By incorporating patient age, site‐specific anatomy, and organ‐specific dose sensitivity, clinicians can implement evidence‐informed imaging protocols that minimize harm while preserving accuracy, particularly crucial in curative‐intent treatments.

## 5 CONCLUSION

This study demonstrates that while MV‐CBCT imaging contributes a measurable dose to organs‐at‐risk, the associated NTCP remains minimal across all evaluated sites. However, secondary cancer risk, as modeled through EAR, reveals more substantial variations, particularly among younger breast cancer patients exposed to 10 MU imaging. This divergence underscores the importance of moving beyond standardized imaging protocols.

By integrating dosimetric data with biologically based risk models, we provide evidence supporting the development of risk‐adaptive CBCT strategies. Tailoring imaging frequency and dose based on tumor site, patient age, and OAR sensitivity can help minimize unnecessary harm without compromising image‐guided precision. This approach is especially relevant in curative settings where long‐term survivorship and secondary malignancy risks are of concern.

Our findings support the implementation of personalized CBCT protocols as part of a broader patient‐centered radiotherapy framework. Future studies may expand on these results by incorporating multi‐institutional cohorts, evaluating alternative imaging modalities, and refining model inputs using prospective outcomes.

## AUTHOR CONTRIBUTIONS

Duong Thanh Tai, Parham Alaei, David Bradley, and James C. L. Chow: *Conceptualization, methodology, data simulation and collection, and writing—original draft*. Tran Trung Kien, Nguyen Ngoc Anh, Luong Tien Phat, and Duong Tuan Linh: *Formal analysis, visualization, and data simulation and collection*. Nguyen Quang Hung, Duong Thanh Tai, Peter Sandwall, Parham Alaei, David Bradley, and James C. L. Chow: *Review, revision, and final approval of the manuscript*.

## CONFLICT OF INTEREST STATEMENT

The authors declare no conflict of interest.

## Supporting information



Supporting Information
