# Supplementary material for: From standard to stratified: Modeling NTCP and EAR to personalize daily MV‐CBCT in radiotherapy
Source: J Appl Clin Med Phys. 2025 Dec 10;26(12):e70414. doi: 10.1002/acm2.70414 (PMC12695683; doi:10.1002/acm2.70414)
Supplement: Supplementary file 1 — Supporting Information [file ACM2-26-e70414-s001.docx]

**Supplementary**

# Supplementary Table 1 – Patient Information Summary

| **No** | **Cancer Type** | **Sex** | **Age at treatment** |
| --- | --- | --- | --- |
| 1 | breast_left | F | 63 |
| 2 | breast_left | F | 54 |
| 3 | breast_left | F | 50 |
| 4 | breast_left | F | 34 |
| 5 | breast_left | F | 64 |
| 6 | breast_left | F | 47 |
| 7 | breast_left | F | 62 |
| 8 | breast_left | F | 66 |
| 9 | breast_left | F | 49 |
| 10 | breast_left | F | 65 |
| 11 | breast_left | F | 76 |
| 12 | breast_left | F | 61 |
| 13 | breast_left | F | 59 |
| 14 | breast_left | F | 44 |
| 15 | breast_left | F | 52 |
| 16 | breast_left | F | 52 |
| 17 | breast_left | F | 63 |
| 18 | breast_left | F | 59 |
| 19 | breast_left | F | 55 |
| 20 | breast_left | F | 51 |
| 21 | breast_left | F | 60 |
| 22 | breast_left |  | 59 |
| 23 | breast_left | F | 65 |
| 24 | breast_left | F | 59 |
| 25 | breast_left | F | 71 |
| 26 | breast_left | F | 55 |
| 27 | breast_left | F | 38 |
| 28 | breast_left | F | 42 |
| 29 | breast_left | F | 68 |
| 30 | breast_right | F | 41 |
| 31 | breast_right | F | 68 |
| 32 | breast_right | F | 72 |
| 33 | breast_right | F | 37 |
| 34 | breast_right | F | 71 |
| 35 | breast_right | F | 44 |
| 36 | breast_right | F | 52 |
| 37 | breast_right | F | 64 |
| 38 | breast_right | F | 45 |
| 39 | breast_right | F | 60 |
| 40 | breast_right | F | 53 |
| 41 | breast_right | F | 57 |
| 42 | breast_right | F | 50 |
| 43 | breast_right | F | 43 |
| 44 | breast_right | F | 59 |
| 45 | breast_right | F | 39 |
| 46 | breast_right | F | 46 |
| 47 | breast_right | F | 58 |
| 48 | breast_right | F | 57 |
| 49 | breast_right | F | 59 |
| 50 | breast_right | F | 43 |
| 51 | breast_right | F | 36 |
| 52 | breast_right | F | 51 |
| 53 | breast_right | F | 43 |
| 54 | breast_right | F | 55 |
| 55 | breast_right | F | 63 |
| 56 | breast_right | F | 73 |
| 57 | breast_right | F | 31 |
| 58 | breast_right | F | 48 |
| 59 | HeadNeck_CBCT | M | 63 |
| 60 | HeadNeck_CBCT | F | 68 |
| 61 | HeadNeck_CBCT | M | 75 |
| 62 | HeadNeck_CBCT | M | 61 |
| 63 | HeadNeck_CBCT | F | 72 |
| 64 | HeadNeck_CBCT | F | 46 |
| 65 | HeadNeck_CBCT | M | 58 |
| 66 | HeadNeck_CBCT | F | 70 |
| 67 | HeadNeck_CBCT | M | 53 |
| 68 | HeadNeck_CBCT | M | 63 |
| 69 | HeadNeck_CBCT | M | 48 |
| 70 | HeadNeck_CBCT | F | 37 |
| 71 | HeadNeck_CBCT | F | 44 |
| 72 | HeadNeck_CBCT | M | 65 |
| 73 | HeadNeck_CBCT | M | 41 |
| 74 | HeadNeck_CBCT | M | 82 |
| 75 | HeadNeck_CBCT | M | 64 |
| 76 | HeadNeck_CBCT | M | 58 |
| 77 | HeadNeck_CBCT | M | 72 |
| 78 | HeadNeck_CBCT | M | 59 |
| 79 | HeadNeck_CBCT | F | 48 |
| 80 | HeadNeck_CBCT | F | 23 |
| 81 | HeadNeck_CBCT | F | 60 |
| 82 | HeadNeck_CBCT | M | 56 |
| 83 | HeadNeck_CBCT | M | 17 |
| 84 | HeadNeck_CBCT | M | 71 |
| 85 | HeadNeck_CBCT | F | 63 |
| 86 | HeadNeck_CBCT | F | 73 |
| 87 | HeadNeck_CBCT | M | 64 |
| 88 | HeadNeck_CBCT | F | 79 |
| 89 | HeadNeck_CBCT | M | 63 |
| 90 | HeadNeck_CBCT | M | 62 |
| 91 | HeadNeck_CBCT | M | 40 |
| 92 | HeadNeck_CBCT | M | 49 |
| 93 | HeadNeck_CBCT | M | 54 |
| 94 | HeadNeck_CBCT | M | 42 |
| 95 | HeadNeck_CBCT | M | 61 |
| 96 | HeadNeck_CBCT | M | 68 |
| 97 | HeadNeck_CBCT | F | 42 |
| 98 | HeadNeck_CBCT | M | 60 |
| 99 | HeadNeck_CBCT | M | 52 |
| 100 | HeadNeck_CBCT | M | 55 |
| 101 | HeadNeck_CBCT | M | 77 |
| 102 | HeadNeck_CBCT | M | 50 |
| 103 | HeadNeck_CBCT | M | 71 |
| 104 | HeadNeck_CBCT | M | 49 |
| 105 | HeadNeck_CBCT | F | 45 |
| 106 | HeadNeck_CBCT | F | 35 |
| 107 | HeadNeck_CBCT | F | 30 |
| 108 | HeadNeck_CBCT | M | 40 |
| 109 | HeadNeck_CBCT | F | 60 |
| 110 | HeadNeck_CBCT | M | 53 |
| 111 | HeadNeck_CBCT | M | 51 |
| 112 | HeadNeck_CBCT | M | 65 |
| 113 | HeadNeck_CBCT | M | 62 |
| 114 | Pelvis_CBCT | M | 72 |
| 115 | Pelvis_CBCT | F | 65 |
| 116 | Pelvis_CBCT | M | 66 |
| 117 | Pelvis_CBCT | M | 59 |
| 118 | Pelvis_CBCT | M | 69 |
| 119 | Pelvis_CBCT | M | 63 |
| 120 | Pelvis_CBCT | M | 64 |
| 121 | Pelvis_CBCT | F | 58 |
| 122 | Pelvis_CBCT | F | 47 |
| 123 | Pelvis_CBCT | M | 60 |
| 124 | Pelvis_CBCT | F | 72 |
| 125 | Pelvis_CBCT | M | 48 |
| 126 | Pelvis_CBCT | M | 52 |
| 127 | Pelvis_CBCT | M | 24 |
| 128 | Pelvis_CBCT | M | 62 |
| 129 | Pelvis_CBCT | F | 57 |
| 130 | Pelvis_CBCT | M | 75 |
| 131 | Pelvis_CBCT | M | 59 |
| 132 | Pelvis_CBCT | F | 79 |
| 133 | Pelvis_CBCT | F | 30 |
| 134 | Pelvis_CBCT | M | 73 |
| 135 | Pelvis_CBCT | M | 45 |
| 136 | Pelvis_CBCT | M | 67 |
| 137 | Pelvis_CBCT | F | 47 |
| 138 | Pelvis_CBCT | M | 70 |
| 139 | Pelvis_CBCT | F | 61 |
| 140 | Pelvis_CBCT | M | 71 |
| 141 | Pelvis_CBCT | F | 72 |
| 142 | Pelvis_CBCT | M | 48 |
| 143 | Pelvis_CBCT | F | 67 |
| 144 | Pelvis_CBCT | M | 67 |
| 145 | Pelvis_CBCT | M | 69 |
| 146 | Pelvis_CBCT | F | 66 |
| 147 | Pelvis_CBCT | M | 45 |
| 148 | Pelvis_CBCT | M | 57 |
| 149 | Pelvis_CBCT | F | 57 |
| 150 | Pelvis_CBCT | M | 76 |
| 151 | Pelvis_CBCT | F | 31 |
| 152 | Pelvis_CBCT | M | 48 |
| 153 | Pelvis_CBCT | F | 59 |
| 154 | Pelvis_CBCT | M | 69 |
| 155 | Pelvis_CBCT | M | 59 |
| 156 | Pelvis_CBCT | M | 76 |

**Supplementary Table 2**: Normal Tissue Complication Probability (NTCP) model parameters (This table summarizes the NTCP model parameters used for three cancer sites (breast, pelvic, and head & neck) in the current study. The values include the NTCP model type and specific parameters such as D50, m, and n or γ, extracted from relevant literature references.

| **Organ** | **Parameters** | **References** |
| --- | --- | --- |
| Breast_CNTR | D50=30.89 Gy, γ=1.3 | [39] |
| Heart | D50=48 Gy, m=0.1, n=0.35 | [40] |
| Lung_IPSI/CNTR | D50=24.5 Gy, m=0.35, n=0.87 | [41] |
| Bladder | D50=80 Gy, m=0.15, n=0.5 | [42] |
| Bowel_Bag | D50=50 Gy, m=0.2, n=0.15 | [43] |
| Femur_L/Femur_R | D50=60 Gy, m=0.2, n=0.3 | [44] |
| Rectum | D50=80 Gy, m=0.09, n=0.09 | [45] |
| Brainstem | D50=54 Gy, m=0.15, n=0.1 | [46] |
| Chiasm | D50=54 Gy, m=0.15, n=0.1 | [47] |
| Optic nerves | D50=55 Gy, m=0.15, n=0.1 | [48] |
| Parotid glands | D50=39.9 Gy, m=0.4, n=1.0 | [49] |
| Eyes & Lenses | D50=8 Gy, m=0.3, n=0.01 | [50] |

Supplementary Table 3: EAR modeling parameters

| **Organ** | **Cancer Type** | **Model** | **Parameters Used** | **References** |
| --- | --- | --- | --- | --- |
| Breast_CNTR | Breast | OED-based | α′=0.085, EAR₀=9.2, γₑ=–0.3, γₐ=1.0 | [27,51] |
| Lung_IPSI | Breast | OED-based | α′=0.085, EAR₀=9.6, γₑ=–0.3, γₐ=1.0 | [27,51] |
| Lung_CNTR | Breast | OED-based | α′=0.085, EAR₀=9.6, γₑ=–0.3, γₐ=1.0 | [27,51] |
| Heart | Breast | OED-based | α′=0.085, EAR₀=0.3, γₑ=–0.3, γₐ=1.0 | [27,51] |
| Bladder | Pelvic | OED-based | α′=0.085, EAR₀=2.1, γₑ=–0.3, γₐ=1.0 | [27,51] |
| Rectum | Pelvic | OED-based | α′=0.085, EAR₀=2.0, γₑ=–0.3, γₐ=1.0 | [27,51] |
| Bowel_Bag | Pelvic | OED-based | α′=0.085, EAR₀=2.0, γₑ=–0.3, γₐ=1.0 | [27,51] |
| Brainstem | Head & Neck | OED-based | α′=0.085, EAR₀=0.6, γₑ=–0.3, γₐ=1.0 | [27,51] |
| SpinalCord+1mm | Head & Neck | OED-based | α′=0.085, EAR₀=0.5, γₑ=–0.3, γₐ=1.0 | [27,51] |
| Parotid_L/R | Head & Neck | OED-based | α′=0.085, EAR₀=0.8, γₑ=–0.3, γₐ=1.0 | [27,51] |
| OpticNerve_L/R | Head & Neck | OED-based | α′=0.085, EAR₀=0.7, γₑ=–0.3, γₐ=1.0 | [27,51] |

## Supplementary Table 4: Mean EAR by Age Group, ROI, and CBCT Plan

(EAR values are shown as cases per 10,000 person-years [×10⁻⁴ / PY])

| Age Group | ROI | Plan | Mean EAR (×10⁻⁴ / PY) | Std. Dev. | Median EAR |
| --- | --- | --- | --- | --- | --- |
| 40-60 | Breast_CNTR | 10MU | 0.1765 | 0.2942 | 0.0370 |
| 40-60 | Breast_CNTR | 5MU | 0.1609 | 0.2718 | 0.0317 |
| 40-60 | Lung_CNTR | 10MU | 0.1769 | 0.2773 | 0.0420 |
| 40-60 | Lung_CNTR | 5MU | 0.1586 | 0.2500 | 0.0383 |
| 40-60 | Lung_IPSI | 10MU | 0.2097 | 0.3256 | 0.0470 |
| 40-60 | Lung_IPSI | 5MU | 0.2171 | 0.3367 | 0.0490 |
| <40 | Breast_CNTR | 10MU | 3.7322 | 2.6965 | 3.5762 |
| <40 | Breast_CNTR | 5MU | 3.3431 | 2.3254 | 3.2615 |
| <40 | Lung_CNTR | 10MU | 7.4572 | 7.4118 | 4.4350 |
| <40 | Lung_CNTR | 5MU | 6.7526 | 6.7202 | 4.0039 |
| <40 | Lung_IPSI | 10MU | 8.7805 | 8.9221 | 5.0177 |
| <40 | Lung_IPSI | 5MU | 9.0918 | 9.2400 | 5.2141 |
| >60 | Breast_CNTR | 10MU | 0.0008 | 0.0007 | 0.0008 |
| >60 | Breast_CNTR | 5MU | 0.0007 | 0.0007 | 0.0007 |
| >60 | Lung_CNTR | 10MU | 0.0009 | 0.0008 | 0.0008 |
| >60 | Lung_CNTR | 5MU | 0.0008 | 0.0007 | 0.0007 |
| >60 | Lung_IPSI | 10MU | 0.0010 | 0.0009 | 0.0010 |
| >60 | Lung_IPSI | 5MU | 0.0011 | 0.0010 | 0.0010 |

**Supplementary Table 5:** Summary of EAR for Breast Cancer Patients by ROI and CBCT Plan (Mean ± SD, Median, IQR).

| ROI | Plan | mean | std | median | Q1 | Q3 | IQR |
| --- | --- | --- | --- | --- | --- | --- | --- |
| Breast_CNTR | 10MU | 0.5 | 1.4 | 0.009 | 0.0009 | 0.1 | 0.1 |
| Breast_CNTR | 5MU | 0.4 | 1.2 | 0.008 | 0.0008 | 0.1 | 0.1 |
| Lung_CNTR | 10MU | 0.9 | 3.2 | 0.016 | 0.0017 | 0.2 | 0.2 |
| Lung_CNTR | 5MU | 0.8 | 2.9 | 0.014 | 0.0016 | 0.2 | 0.2 |
| Lung_IPSI | 10MU | 1.0 | 3.8 | 0.020 | 0.0020 | 0.3 | 0.3 |
| Lung_IPSI | 5MU | 1.1 | 3.9 | 0.020 | 0.0020 | 0.3 | 0.3 |

**Supplementary Table A6**. Summary of Excess Absolute Risk (EAR) for Pelvis Region by ROI and CBCT Plan (Mean ± SD, Median, IQR).

| ROI | Plan | mean | std | median | Q1 | Q3 | IQR |
| --- | --- | --- | --- | --- | --- | --- | --- |
| Bladder | 10MU | 0.2 | 1.1 | 0.00011 | 0.000011 | 0.0012 | 0.0012 |
| Bladder | 5MU | 0.3 | 1.2 | 0.00011 | 0.000012 | 0.0013 | 0.0013 |
| Bowel_Bag | 10MU | 0.5 | 3.1 | 0.00008 | 0.000013 | 0.0008 | 0.0008 |
| Bowel_Bag | 5MU | 0.6 | 3.2 | 0.00009 | 0.000013 | 0.0008 | 0.0008 |
| Rectum | 10MU | 0.1 | 0.5 | 0.00005 | 0.000004 | 0.0006 | 0.0006 |
| Rectum | 5MU | 0.1 | 0.5 | 0.00005 | 0.000004 | 0.0006 | 0.0006 |

**Supplementary Table A7**. Summary of Excess Absolute Risk (EAR) for **Head and Neck** Region by ROI and CBCT Plan (Mean ± SD, Median, IQR).

| ROI | Plan | mean | std | median | Q1 | Q3 | IQR |
| --- | --- | --- | --- | --- | --- | --- | --- |
| Brainstem | 10MU | 2.07 | 13.01 | 0.0003 | 0.00003 | 0.005 | 0.005 |
| Brainstem | 5MU | 2.13 | 13.35 | 0.0003 | 0.00003 | 0.005 | 0.005 |
| OpticNerve_L | 10MU | 2.72 | 17.34 | 0.0003 | 0.00002 | 0.006 | 0.006 |
| OpticNerve_L | 5MU | 2.67 | 16.93 | 0.0002 | 0.00001 | 0.006 | 0.006 |
| OpticNerve_R | 10MU | 2.45 | 16.71 | 0.0003 | 0.00001 | 0.006 | 0.006 |
| OpticNerve_R | 5MU | 2.51 | 17.11 | 0.0003 | 0.00001 | 0.006 | 0.006 |
| Parotid_L | 10MU | 0.09 | 0.48 | 0.0002 | 0.00006 | 0.004 | 0.004 |
| Parotid_L | 5MU | 0.09 | 0.48 | 0.0003 | 0.00006 | 0.005 | 0.005 |
| Parotid_R | 10MU | 0.03 | 0.11 | 0.0002 | 0.00005 | 0.006 | 0.006 |
| Parotid_R | 5MU | 0.03 | 0.11 | 0.0002 | 0.00006 | 0.006 | 0.006 |
| SpinalCord+1mm | 10MU | 0.54 | 2.16 | 0.0001 | 0.00002 | 0.004 | 0.004 |
| SpinalCord+1mm | 5MU | 0.49 | 1.97 | 0.0001 | 0.00002 | 0.004 | 0.004 |
